# Profil épidémiologique de la coqueluche du nourrisson à Casablanca de 2012 à 2019

**DOI:** 10.11604/pamj.2023.46.124.42073

**Published:** 2023-12-29

**Authors:** Bouchra Slaoui, Hajar Saidi, Meryem Kamal, Khalid Kafty, Jalal Nourlil, Idrissa Diawara, Khalid Zerouali, Houria Belabbes, Naima Elmdaghri

**Affiliations:** 1 Département de Pédiatrie, Faculté de Médecine et de Pharmacie, Université Hassan II, Casablanca, Maroc,; 2 Unité de Pneumo-allergologie Pédiatrique, Service de Pédiatrie 2, Hôpital Mère-Enfants Abderrahim Harouchi, Centre Hospitalier Universitaire Ibn Rochd, Casablanca, Maroc,; 3 Département de Microbiologie, Faculté de Médecine et de Pharmacie, Université Hassan II, Casablanca, Maroc,; 4 Service de Microbiologie, Centre Hospitalier Universitaire Ibn Rochd, Casablanca, Maroc,; 5 Laboratoire de Virologie, Institut Pasteur du Maroc, Casablanca,; 6 Faculté des Sciences et Techniques de la Santé, Université Mohamed VI des Sciences de la Santé, Casablanca, Maroc

**Keywords:** Coqueluche, nourrisson, épidémiologie, *Bordetella Pertussis*, Whooping cough, Pertussis, infant, epidemiology, Bordetella Pertussis

## Abstract

La coqueluche est un véritable problème de santé publique en raison de sa morbidité importante chez le jeune nourrisson et sa résurgence malgré une couverture vaccinale élevée. Le but de cette étude est d´analyser le profil épidémiologique de la coqueluche des nourrissons hospitalisés de 2012 à 2019. C´est une étude rétrospective, descriptive menée sur une période de 7 ans et 8 mois, de janvier 2012 à juillet 2019. Elle a concerné 500 nourrissons admis pour une suspicion clinique de coqueluche. L´âge moyen était de 72 jours avec des extrêmes de 28 jours et 18 mois. Soixante-quinze pour cent (75%) des nourrissons avaient moins de 3 mois. Le pic d´incidence a été noté en 2012 et 2016 avec une prédominance estivale (32%). Quatre cent soixante (460) nourrissons (92%) étaient non ou incomplètement vaccinés dont 42,2% étaient trop jeunes pour l´être. Un contaminateur probable dans l´entourage a été retrouvé dans 43,6% des cas. La toux quinteuse cyanosante était le principal motif d´hospitalisation (77,6%). La radiographie pulmonaire avait objectivé un syndrome bronchique (25,4%) et des foyers alvéolaires dans (22,7%) des cas. L´hémogramme réalisé chez 410 nourrissons avait retrouvé une hyperlymphocytose dans 67,5 % des cas. La réaction en chaine par polymérase (PCR) sur prélèvement nasopharyngé réalisée chez 206 nourrissons, était positive pour Bordetella pertussis dans 64% des cas. Cent dix-huit (118) PCR effectuées chez les mères étaient positives dans 47,7% des cas. Tous les nourrissons ont été mis sous Clarithromycine. La coqueluche est une cause importante de morbidité chez le nourrisson à Casablanca. La prévention repose sur la vaccination de tout l´entourage familial des jeunes nourrissons. Cependant la vaccination des femmes enceintes semble plus efficace.

## Abstract (en)

Pertussis is a real public health problem due to high neonatal morbidity rates and resurgence despite high vaccination coverage. The purpose of this study is to analyze the epidemiological profile of pertussis in infants hospitalized from 2012 to 2019. We conducted a retrospective, descriptive study over a 7-year and 8-month period from January 2012 to July 2019. It involved 500 infants admitted with clinical suspicion of pertussis. The average age of infants was 72 days, ranging from 28 days to 18 months; 75% of infants were less than 3 months old. The peak incidence was registered in 2012 and 2016, with a summer predominance (32%); 460 infants (92%) were not or incompletely vaccinated, 42.2% of whom were too young to be vaccinated. A probable contaminant in the entourage was found in 43,6% of cases. Whooping cough and cyanosis were the main reason for hospitalization (77.6%). Chest radiography objectified bronchial disease (25,4%) and alveolar foci (22.7%). Blood count performed in 410 infants showed hyperlymphocytosis in 67.5% of cases. Polymerase chain reaction (PCR) on nasopharyngeal sample collected from 206 infants was positive for Bordetella pertussis in 64% of cases; 118 PCR performed in mothers were positive in 47.7% of cases. All infants received Clarithromycin. Pertussis is a major cause of morbidity in infants in Casablanca. The prevention strategy is based on vaccination of family members of infants. However, vaccination of pregnant women appears to be more effective.

## Introduction

La coqueluche demeure un problème de santé publique à l´échelle mondiale et l´une des maladies évitables par la vaccination, les moins contrôlées au monde [1]. Il s´agit d´une maladie endémique cyclique qui connait des pics d´activités tous les 2 à 5 ans [2]. Le diagnostic clinique de la coqueluche reste facile dans ses formes les plus typiques du jeune nourrisson non immunisé. Du fait de la vaccination, les formes atypiques sont devenues les plus fréquentes et touchent les grands enfants, adolescents et adultes. Ces formes cliniques passent inaperçues et contribuent à la dissémination de l´infection et la contamination de très jeunes nourrissons qui sont sujet aux complications parfois létales [3]. Dans la plupart des cas le diagnostic reste clinique pour ne pas retarder la prise en charge médicale par l´instauration d´un traitement précoce afin de prévenir la contagiosité et les complications [4]. Après la généralisation de la vaccination, ce sont essentiellement les jeunes nourrissons non ou incomplètement vaccinés qui sont contaminés par les adolescents et les jeunes adultes [5]. Cette étude a pour objectifs d´analyser le profil épidémiologique, clinique et bactériologique des cas de coqueluche hospitalisés de janvier 2012 à juillet 2019.

## Méthodes

**Conception de l´étude**: c´est une étude rétrospective descriptive de tous les cas de coqueluche du nourrisson hospitalisés de janvier 2012 à juillet 2019.

**Cadre de l´étude et population**: cette étude a été réalisée dans l´unité de pneumo-allergologie pédiatrique de l´Hôpital Abderrahim Harrouchi à Casablanca. Nous avons inclus tous les nourrissons admis pour une suspicion clinique de coqueluche devant: des quintes de toux spasmodiques et/ou cyanosantes et émétisantes, associées ou non à une reprise inspiratoire bruyante en « chant de coq », avec ou sans confirmation de la coqueluche par réaction en chaine par polymérase (PCR) ou par l´isolement de *Bordetella pertussis* à la culture. Nous avons inclus également les nourrissons hospitalisés pour des quintes de toux spasmodique cyanosante avec reprise inspiratoire en chant de coq et sibilants à l´auscultation par crainte d´une co-infection virale.

**Outils de collecte de données**: les dossiers ont été analysés selon des fiches d´exploitation préalablement établies. La saisie informatique a été réalisée à l´aide de « Microsoft Excel version 2010 ». L´analyse statistique et le traitement des données ont été menés à l´aide du logiciel SPSS.

**Collecte de données**: les variables recueillies sont l´âge, le sexe, le statut vaccinal, la présence d´un tousseur dans l´entourage et le mode de garde du nourrisson. Les autres données précisées concernent le tableau clinique: les caractéristiques de la toux, la présence d´une cyanose, d´un chant de coq et d´apnées. L´examen clinique a précisé les données de l´auscultation pulmonaire. Le bilan réalisé concerne la radiographie pulmonaire, la numération et formule sanguine et la PCR sur prélèvement nasal réalisées chez les nourrissons et leurs mères.

**Analyse statistique**: les données ont été saisies et analysées sur tableur Excel. Les données quantitatives ont été exprimées en pourcentage, moyenne, médiane et écart type.

## Résultats

**Figure 1:**
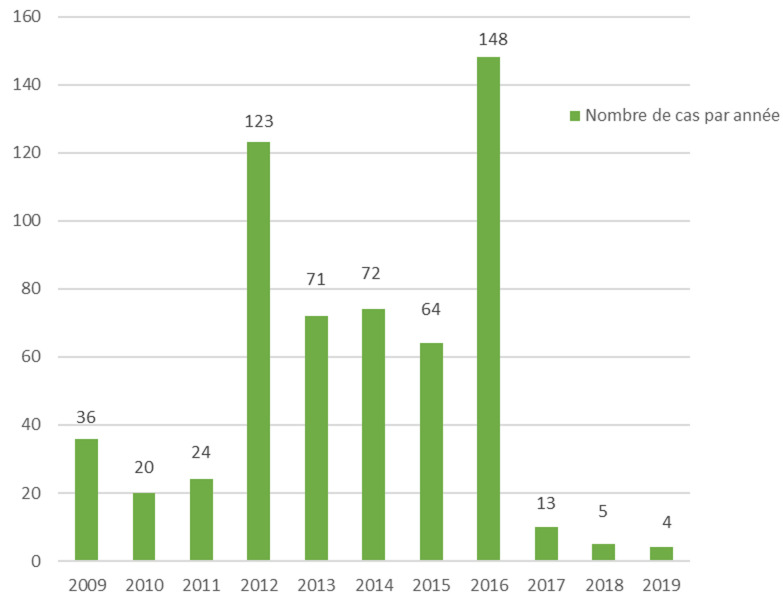
répartition des cas de coqueluche selon les années: deux pics épidémiques en 2012 et en 2019

**Figure 2:**
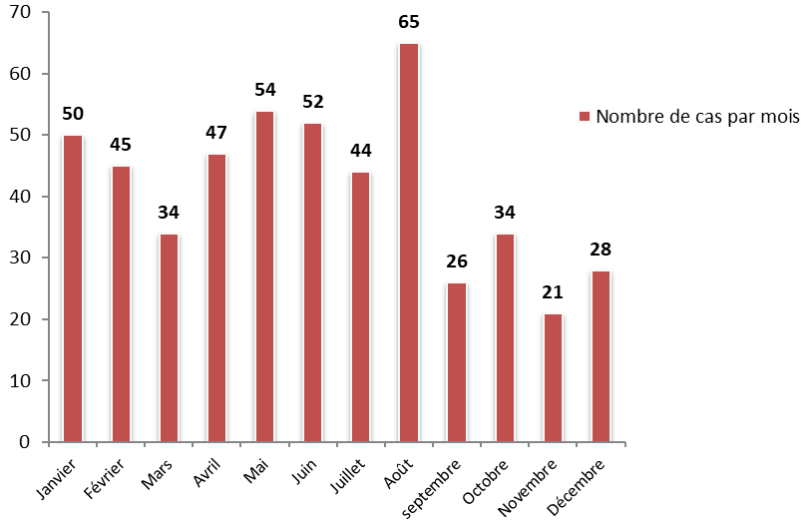
répartition mensuelle des cas de coqueluche du nourrisson de 2012 à 2019

**Profil épidémiologique**: de janvier 2012 à fin juillet 2019, 500 nourrissons ont été hospitalisés pour une suspicion de coqueluche, avec deux pics d´incidence en 2012 et 2016 (Figure 1). Parmi ces nourrissons, 32% soit 161 cas ont été hospitalisés durant la saison estivale (Figure 2). Le sexe ratio était de 1,04 avec 256 garçons (51,2%) et 244 filles (48,8%). L´âge des patients était compris entre 28 jours et 18 mois, avec une moyenne de 72 jours. Parmi ces nourrissons, 357 étaient âgés de moins de 3 mois (75%), 20,2% entre 3 et 6 mois, 3,8% entre 6 mois et 1 an et 1% étaient âgés de plus d´un an. Concernant le statut vaccinal, 460 (92%) nourrissons étaient non ou incomplètement vaccinés: 304 nourrissons (60,8%) étaient non vaccinés dont 42,2% étaient âgés de moins de 2 mois, 114 nourrissons (22,8%) avaient reçu une seule dose de vaccin anticoquelucheux et 42 nourrissons avaient reçu 2 doses de vaccin anticoquelucheux (Tableau 1). Un contaminateur probable, tousseur dans l´entourage a été retrouvé dans 208 cas soit 43,6%. Il s´agit de 119 mères (54,58%) et 10 pères, 26 grand parents et 53 frères et sœurs. Concernant le mode de garde, 347 nourrissons (69.4%) étaient gardés à domicile.

**Tableau 1:** répartition des cas de coqueluche selon le statut vaccinal

Variables			Doses vaccinales			
Age	Effectif	Pourcentage	0	1 dose	2 doses	3 doses
			Nombre (%)	Nombre (%)	Nombre (%)	Nombre (%)
**< 2 mois**	211	42,20%	211(42,2%)	0(0%)	0(0%)	0(0%)
**≥2 mois à 3 mois**	164	32,80%	81(16,2%)	83(16,6%)	0(0%)	0(0%)
**≥3mois à 4 mois**	72	14,40%	9 (1,8%)	28 (5,6%)	35 (7%)	0 (0%)
**≥ 4 mois**	53	10,60%	3(0,6%)	3(0,6%)	7(1,4%)	40(8%)
**Total**	500	100%	304(60,8%)	114(22,8%)	42 (8,4%)	40(8%)

**Profil clinique**: les nourrissons avaient une toux quinteuse (99,2%), émétisante (62,8%) et cyanosante (91%). Ces quintes étaient suivies de reprise inspiratoire bruyante en chant de coq chez 12,6% des nourrissons, accompagnées d´apnée dans 12,4% des cas. Les quintes de toux étaient responsables d´accès hypoxiques, prolongés et sévères chez 9,2% des nourrissons. Les nourrissons étaient apyrétiques dans 84% des cas. L´auscultation pulmonaire avait objectivé des râles ronflants chez 90 nourrissons (18%), des crépitants chez 74 nourrissons (14,8%) et des râles sibilants chez 76 nourrissons (15,2%). Les râles sibilants avaient été rattachés à une coïnfection avec un virus respiratoire. La radiographie pulmonaire était normale chez 228 nourrissons (51,8%). Elle avait objectivé un syndrome bronchique (25,4%) et des foyers alvéolaires chez 22,7% des enfants. La numération formule sanguine réalisée chez 410 nourrissons (82%) avaient retrouvé une hyperlymphocytose supérieure ou égale à 10000/mm^3^ dans 276 cas (67,5%). Le nombre de lymphocytes était très élevé compris entre 20000 et 40990/mm^3^ chez 10 nourrissons. Sur le plan bactériologique, en 2012 un échantillon de 16 prélèvements naso-pharyngés avait été adressé à l´institut pasteur de Casablanca. La PCR était positive pour *Bordetella pertussis* chez 12 nourrissons. Ces premiers résultats avaient permis de confirmer l´épidémie de 2012. De janvier 2013 à juillet 2019, 190 prélèvements naso-pharyngés ont été effectués chez les nourrissons dans le laboratoire de microbiologie du CHU Ibn Rochd. Après extraction, l'analyse de l'ADN a été effectuée par PCR en temps réel (RT-PCR): L´ADN de *Bordetella pertussis* a été détecté chez 121 nourrissons (64%). Parmi les contacts familiaux, 118 PCR effectuées chez les mères étaient positives dans 47,7% pour *Bordetella pertussis*. Tous les nourrissons ont été mis sous macrolides: Clarithromycine 15mg /kg/j pendant 10 jours. Les mères ont toutes reçu de l´azithromycine 500 mg par jour pendant 3 jours. Les nourrissons qui présentaient une pneumonie associée ou un sepsis avaient été mis sous céphalosporine de 3^e^ génération par voie intraveineuse. Concernant l´évolution, 14 nourrissons âgés de moins de 3 mois ont été transférés en réanimation pour des quintes asphyxiantes (5 cas), une apnée syncopale (7 cas), une détresse respiratoire sévère (2 cas) ou des convulsions (8 cas). L´évolution était favorable dans les autres cas. La durée moyenne de séjour était de 8 jours avec des extrêmes de 5 jours à un mois. Six nourrissons ont été réhospitalisés après leur sortie en raison d´une recrudescence des quintes de toux.

## Discussion

Cette étude montre que la coqueluche est responsable d´une morbidité importante chez le jeune nourrisson non ou incomplètement vacciné. Les épidémies de 2012 et 2016 sont les plus importantes dans notre hôpital ces 15 dernières années. La coqueluche est une cause importante de mortalité infantile dans le monde et reste un problème de santé publique, même dans les pays à forte couverture vaccinale. A l´échelle mondiale, on estime qu´il y avait 24,1 millions de cas de coqueluche et 160700 décès dus à la coqueluche chez les enfants de moins de 5 ans en 2014 [1]. Ce sont les nourrissons de moins de 3 mois qui sont les plus à risque. Le pic d´incidence de la coqueluche retrouvée en 2012 et 2013 à Casablanca est également rapporté en France, en Belgique, au Royaume Uni et aux Etats Unis [6-9]. En France, de 1996 à 2012, 3318 cas de coqueluche ont été confirmés chez les nourrissons âgés de moins de 6 mois [6]. Les taux d'incidence étaient estimés à 179 pour 100 000 nourrissons par an de 2008 à 2012 [7]. En Belgique en 2013 (Wallonie) 409 cas ont été déclarés, le taux de notification est le plus important chez les enfants de moins d´un an avec 129 cas /100000 [8]. Aux Etats-Unis, le *Centrer for Disease Control and Prevention* a rapporté 48277 cas en 2012 [9].

L´épidémiologie de la coqueluche en Afrique est difficile à évaluer en raison de la faiblesse du système de surveillance et de déclaration. En Algérie dans une étude menée entre 2012 et 2013, la coqueluche a été confirmée chez 134 des 248 patients (54%) avec 119 nourrissons âgés de moins de 18 mois. La distribution saisonnière de ces cas confirmés de coqueluche a montré un regroupement dans la période de trois mois de juillet à septembre [66,4%] [10]. En Tunisie, entre 2007 et 2016, l´incidence estimée de la coqueluche dans la région de Tunis était de 134 cas pour 100000 chez les enfants de moins de 5 ans. Deux pics épidémiques ont été observés en 2009 et 2014 [11]. Au Maroc, la coqueluche est une maladie à déclaration obligatoire. Avant la mise en place du programme national d´immunisation en 1985, des milliers de cas étaient enregistrés annuellement [12-14]. 1078 cas de coqueluche ont été déclarés en 1987 avec une couverture vaccinale de 85% au vaccin anticoquelucheux combiné à celui de la diphtérie et du tétanos. Le nombre de cas déclarés a par la suite été réduit à moins de 100 cas par an en 2011 [12]. Le taux d´incidence est passé de 76,9 pour 100000 habitants en 1980 à 0,05 en 2010 [14]. Une résurgence a été observée en 2012 malgré une couverture vaccinale dépassant 95% au Maroc [12,13]. La coqueluche reste une maladie infectieuse endémique mondiale, avec des flambées épidémiques généralement tous les 3 à 5 ans et une saisonnalité été-automne [15]. Ce phénomène est lié essentiellement à un affaiblissement de l´immunité après la vaccination [15]. Les résultats de cette étude Casablancaise montrent que la plupart des nourrissons (75%) étaient âgés de moins de 3 mois, non ou incomplètement vaccinés. Cette tranche d´âge est la plus fréquemment touchée dans toutes les séries [16-18].

Au Maroc, en santé publique la vaccination contre la coqueluche (vaccin coquelucheux à cellules entières associé aux anatoxines diphtérique et tétanique) a été introduite dans le programme national d´immunisation (PNI) au début des années 1980. Actuellement, la stratégie de vaccination marocaine en santé publique, comprend une primo vaccination à l´âge de 2,3, 4 mois et deux rappels à l´âge de 18 mois et 5 ans. La couverture vaccinale contre la coqueluche dépasse 95% à l´âge de 24 mois [19]. Les grands enfants, les adolescents et les adultes sont à l´origine de la contamination des nourrissons non encore immunisés. Parmi les facteurs favorisants, on évoque la baisse progressive de l´immunité vaccinale, l´efficacité partielle, aussi bien du vaccin entier que du vaccin acellulaire et l´absence d´immunité passive d´origine maternelle [20]. Dans cette étude Casablancaise, les principaux contaminateurs étaient les mères. Ces données sont semblables à celles des études précédemment publiées [21]. En France et en Australie, le contaminateur principal était le père ou la mère [22,23]. Aux États-Unis, les frères et les sœurs jouent un rôle important dans la transmission de la coqueluche aux jeunes nourrissons et constituent le principal réservoir de l´infection [24]. Sur le plan clinique, Il est important de noter que la respiration sifflante n´est pas une manifestation de la coqueluche sauf en cas d´infection virale concomitante. Di Camillo *et al*. En Italie ont noté une co-infection virale chez 90 enfants (46,15%). L´agent le plus fréquemment isolé était le rhinovirus (26,15%), suivi par l´adénovirus (7,17%) [25].

L´hyperlymphocytose peut faire évoquer la coqueluche si elle est supérieure à 10 000/mm3 sans être toutefois totalement spécifique ni constante. L´hyperlymphocytose semble être un marqueur de gravité, sans que sa valeur prédictive n´ait été évaluée avec précision [26]. *Bordetella pertussis* est la bactérie de loin la plus souvent identifiée aussi bien dans cette série Casablancaise (96,93%) que dans les autres séries algériennes (100%) [10], Tunisienne (86,6%) [11] et françaises (91,4%) [6]. De nombreuses études ont montré l´importance de la PCR en temps réel dans le diagnostic de la coqueluche. Au Maroc, dans l´étude de Kafty, la détection directe de l'ADN bactérien par des techniques de PCR, notamment la RT- PCR, est plus sensible que la culture et a l´avantage d´être rapide [27]. Sur le plan thérapeutique, la prise en charge vise à réduire les complications et à limiter la contagiosité de la maladie [28]. Les nourrissons de moins de 6 mois doivent en général être hospitalisés en raison du risque de quintes asphyxiantes et d´apnée. Durant la phase paroxystique, lorsque la toux est présente, le traitement antibiotique n´a que peu d´effet sur la maladie elle-même, mais il est recommandé d´utiliser un antibiotique pour limiter la transmission de la bactérie à d´autres personnes vulnérables. Le traitement de référence est la clarithromycine 15 mg/kg en 2 prises quotidiennes fractionnées pendant 7 jours et l´azithromycine (dose de 10 mg/kg par jour pendant 5 jours) [29].

La vaccination représente la meilleure arme de prévention individuelle mais aussi collective, tant chez le nourrisson que chez l´adulte. Les vaccins acellulaires confèrent une protection à court terme comparable à celle des vaccins à germes entiers avec moins de réactions locales et systémiques [30]. Il a été démontré que la protection induite par tous les vaccins contre la coqueluche a tendance à s'atténuer avec le temps [31]. Les différentes stratégies pour protéger les nourrissons sont le rappel vaccinal des adolescents, le cocooning et la vaccination des femmes enceintes. En France, en 1998, un rappel tardif de 11 à 13 ans a été introduit dans le calendrier vaccinal afin de réduire le nombre de cas chez les nourrissons contaminés par les adolescents ou les jeunes adultes [32]. En Colombie-Britannique et au Québec, le programme de rappel du vaccin contre le tétanos, la diphtérie et la coqueluche acellulaire (Tdap) pour les adolescents a réduit de manière significative l'incidence de la coqueluche chez les adolescents de 15 à 19 ans [33]. En 2004, le calendrier vaccinal français a introduit la stratégie du cocooning [32]; il s´agit de vacciner contre la coqueluche avec un vaccin acellulaire les adultes en contact avec de très jeunes nourrissons et les adultes susceptibles de devenir parents dans les mois à venir. Les difficultés associées à la stratégie du cocon ont conduit certains pays à adopter la vaccination des femmes enceintes [34,35]. Le principe repose sur le transfert par voie transplacentaire d´une immunité nouvellement acquise (ou renforcée par un rappel) par la mère pendant la grossesse vers son fœtus. Cette approche permet d´assurer la protection des très jeunes enfants dès la naissance. La vaccination pendant la grossesse est actuellement recommandée dans plusieurs pays tels la France, les Etats-Unis, l´Argentine, la Belgique, Israël, la Nouvelle-Zélande et le Royaume-Uni [36].

**Les limites de cette étude**: c´est un travail rétrospectif avec toutes ses difficultés. La numération formule sanguine n´a pas été réalisée chez tous les nourrissons. Il aurait été intéressant de pratiquer une PCR multiplex chez les nourrissons qui présentaient des sifflements et une toux coqueluchoide, afin de préciser la prévalence des coinfections virus-*Bordetella pertussis*.

## Conclusion

La coqueluche demeure un important problème de santé publique au Maroc, malgré une couverture vaccinale très élevée chez les enfants. L´immunité vaccinale diminue avec le temps, d´où une augmentation de l´incidence de la maladie plus élevée chez les adultes et les nourrissons non ou incomplètement vaccinés. Il est urgent de faire savoir, aussi bien aux professionnels de santé qu´à la population, que la coqueluche n´est pas qu´une maladie pédiatrique et que le meilleur moyen de lutter contre elle est de suivre les nouvelles recommandations vaccinales. La surveillance épidémiologique à travers des réseaux, la prise en charge en milieu hospitalier des nourrissons fragiles, la vaccination des adolescents et des adultes contaminateurs et des femmes enceintes sont les composantes d´un contrôle efficace de la maladie.

### Etat des connaissances sur le sujet

- *La coqueluche est un réel problème de santé publique mondial en raison de sa morbidité élevée et de sa mortalité chez le jeune nourrisson non encore vacciné ou incomplètement vacciné;*
- *C´est une infection qui survient par épidémies cycliques tous les 3 à 5 ans;*
- *Les parents et la fratrie sont les principaux contaminateurs selon les pays et les différentes séries publiées*.

### Contribution de notre étude à la connaissance

- *Il s´agit de la plus grande série marocaine de coqueluche du nourrisson publiée à ce jour;*
- *Cette étude illustre le caractère cyclique de l´épidémie; en effet durant cette période nous avons constaté deux grands pics épidémiques: en 2012 et en 2016; le pic attendu en 2020 ou 2021 n´a pas eu lieu en raison de la pandémie à SARS-CoV2 et des mesures barrières; cependant en 2023 nous assistons à un nouveau pic épidémique de coqueluche chez le nourrisson hospitalisé;*
- *La mère est le principal contaminateur des nourrissons hospitalisés, par conséquent la meilleure stratégie préventive à envisager dans notre pays est la vaccination de la femme enceinte*.
